# Supplementary material for: Enhort: a platform for deep analysis of genomic positions
Source: PeerJ Comput Sci. 2019 Jun 10;5:e198. doi: 10.7717/peerj-cs.198 (PMC7924414; doi:10.7717/peerj-cs.198)
Supplement: Table S1 — Publications that were reviewed to evaluate the applicability of Enhort. The publications were found by searching for full papers with the search string “integration site genome” for homo sapiens later than 2005. Reviews, applications notes and Cas9 specific publications were omitted. Additionally referenced publications from these were searched. All publications that used genomic sites were reviewed and estimated if the analysis and figures are possible to re-create using Enhort. Mostly reproduceable publications are marked with an x in the table. Partly reproduceable publications with (x). Additional information about the type of analysis was gathered and is added to the table. [file peerj-cs-05-198-s001.pdf]

DOI	Random control sites	Altered control sites	Integration statistics	Chromosomal distribution	Gene integration	Motif	Reproducable by Enhort
10.1186/1742-4690-10-90			x				(x)
10.1093/nar/gkt1399	x		x	x		x	x
10.1182/blood-2014-02-553602				x			x
10.1126/science.1256304					x		x
10.1016/j.cell.2015.01.020					x	x	x
10.1093/nar/gku769	x		x				x
10.1128/JVI.01148-12			x				x
10.1126/science.1254194					x		
10.1128/JVI.01617-15.			x		x		x
10.1016/j.jcv.2013.12.006							
10.1128/JVI.01135-13				x			(x)
10.1128/MCB.00670-12		x	x		x	x	x
10.1128/JVI.01356-14		x	x	x	x		x
10.1016/j.cell.2013.02.032			x			x	(x)
10.1016/j.cell.2013.09.020			x				(x)
10.1371/journal.pone.0066693							
10.1093/nar/gku136						x	
10.1128/JVI.00011-14		x	x				x
10.1371/journal.pgen.1004250		x	x				x
10.1101/gr.163659.113							
10.1089/hum.2012.112							
10.1016/j.chom.2014.09.016							
10.1186/s12977-015-0145-9							
10.1128/JVI.02995-13							
10.1371/journal.pone.0088718					x		(x)
10.3390/genes5020415	x		x				x
10.1038/mt.2016.52	x			x			
10.3390/cancers7040887	x			x			x
10.1016/j.virol.2013.02.022			x				x
10.18632/oncotarget.6809				x			x
10.1038/mt.2009.94.	x		x	x		x	x
10.1186/1742-4690-6-114						x	(x)
10.1186/s12864-017-3481-4							(x)
10.1089/hum.2010.177	x		x				x
10.1089/hum.2009.134			x	x			x
10.1073/pnas.1001402107							
10.1101/gr.3421505							

DOI	Random control sites	Altered control sites	Integration statistics	Chromosomal distribution	Gene integration	Motif	Reproduceable by Enhort
10.1186/s12977-014-0090-z							
10.1128/JVI.03419-13	x		x	x	x	x	x
10.1371/journal.pone.0004211				x	x		x
10.1101/gr.134395.111							
10.1093/nar/gku175							x
10.1038/mt.2009.16			x	x		x	x
10.1073/pnas.1307157110	x				x		x
10.1371/journal.pbio.0020423	x		x				x
10.1128/JVI.00635-11		x	x				x
10.1128/JVI.78.21.11656-11663.2004	x		x	x			x
10.1038/mt.2016.11	x		x	x			x
10.1101/cshperspect.a006890				x			x
10.1101/gr.6286907		x	x	x		x	x
10.1371/journal.ppat.1001313	x		x		x		x
10.1186/1742-4690-9-84	x		x		x		x
10.1371/journal.ppat.1000985	x		x	x			x
10.1371/journal.ppat.1004117				x			
10.1016/j.jviromet.2013.01.004	x		x				x
10.1371/journal.pbio.0020234	x		x	x			x
10.1371/journal.pcbi.0020157							
10.1126/science.1083413	x		x				x
10.1099/vir.0.81554-0			x	x	x	x	x
10.1016/j.bbrc.2006.05.007	x		x	x	x		x
